# Glypican-3 targeted delivery of ^89^Zr and ^90^Y as a theranostic radionuclide platform for hepatocellular carcinoma

**DOI:** 10.1038/s41598-021-82172-w

**Published:** 2021-02-12

**Authors:** Kevin P. Labadie, Andrew D. Ludwig, Adrienne L. Lehnert, Donald K. Hamlin, Aimee L. Kenoyer, Kevin M. Sullivan, Sara K. Daniel, Tara N. Mihailovic, Jonathan G. Sham, Johnnie J. Orozco, Raymond S. Yeung, Delphine L. Chen, D. Scott Wilbur, Robert S. Miyaoka, James O. Park

**Affiliations:** 1 Department of Surgery, University of Washington Medical Center, 1959 NE Pacific Street, Box 356410, Health Sciences Bldg. Room BB-442, Seattle, WA 98195-6410 USA; 2 Department of Radiology, University of Washington, Seattle, WA USA; 3 Department of Radiation Oncology, University of Washington, Seattle, WA USA; 4 Clinical Research Division, Fred Hutch Cancer Research Center, Seattle, WA USA

**Keywords:** Hepatocellular carcinoma, Molecular medicine

## Abstract

Glypican-3 (GPC3) is a tumor associated antigen expressed by hepatocellular carcinoma (HCC) cells. This preclinical study evaluated the efficacy of a theranostic platform using a GPC3-targeting antibody αGPC3 conjugated to zirconium-89 (^89^Zr) and yttrium-90 (^90^Y) to identify, treat, and assess treatment response in a murine model of HCC. A murine orthotopic xenograft model of HCC was generated. Animals were injected with ^89^Zr-labeled αGPC3 and imaged with a small-animal positron emission/computerized tomography (PET/CT) imaging system (immuno-PET) before and 30 days after radioimmunotherapy (RIT) with ^90^Y-labeled αGPC3. Serum alpha fetoprotein (AFP), a marker of tumor burden, was measured. Gross tumor volume (GTV) and SUV_max_ by immuno-PET was measured using fixed intensity threshold and manual segmentation methods. Immuno-PET GTV measurements reliably quantified tumor burden prior to RIT, strongly correlating with serum AFP (*R*^2^ = 0.90). Serum AFP was significantly lower 30 days after RIT in ^90^Y-αGPC3 treated animals compared to those untreated (*p* = 0.01) or treated with non-radiolabeled αGPC3 (*p* = 0.02). Immuno-PET GTV measurements strongly correlated with tumor burden after RIT (*R*^2^ = 0.87), and GTV of animals treated with ^90^Y-αGPC3 was lower than in animals who did not receive treatment or were treated with non-radiolabeled αGPC3, although this only trended toward statistical significance. A theranostic platform utilizing GPC3 targeted ^89^Zr and ^90^Y effectively imaged, treated, and assessed response after radioimmunotherapy in a GPC3-expressing HCC xenograft model.

## Introduction

Hepatocellular carcinoma (HCC) is the most common histologic subtype of liver cancer with forecasts projecting an increase in incidence in coming decades^1–3^. HCC most commonly presents in the setting of chronic liver disease, often in an advanced stage involving multiple diseased liver segments^4,5^. In cases of advanced HCC, overall survival is poor with the standard-of-care systemic therapy (e.g. sorafenib, lenvatinib) and checkpoint inhibition immunotherapy (e.g. nivolumab, pembrolizumab) only providing marginal survival benefit^6–10^. Novel therapies that combine immune checkpoint inhibitors with anti-angiogenic agents (e.g. atezolizumab and bevacizumab) or tyrosine kinase inhibitor (e.g. pembrolizumab and lenvatitinib) have recently demonstrated promise in advanced HCC, although there are limiting side effects^11–14^. Locoregional therapies, such as transarterial chemoembolization and radioembolization, are important tools in management of locoregional and advanced HCC. They have been shown to prolong survival in unresectable HCC and can facilitate downstaging to transplant or surgical resection^15–17^. Surgical resection is a mainstay of therapy in patients with resectable disease and preserved liver function, however recurrence in the liver remnant is common due to de novo carcinogenesis, intrahepatic metastasis, and missed lesions during initial staging^18^. Therefore, improved diagnostic and therapeutic adjuncts are needed to better stage and treat patients with HCC.

Targeted radionuclide theranostics is an emerging field that promises new and personalized approaches to cancer therapy. To date, numerous targeted radionuclide diagnostic and therapeutic technologies are in development, including positron-emitting diagnostic agents for targeted PET imaging and beta- and alpha-emitting agents as radionuclide therapies, or radioimmunotherapy (RIT). Somatostatin receptor (SSTR) targeted Galium-68 (^68^ Ga) DOTATATE radiopeptide imaging and Lutetium-177 (^177^Lu) DOTATATE peptide receptor radionuclide therapy (PRRT) compose the clinical theranostic platform available for the management of advanced midgut neuroendocrine tumors^19–23^. Currently, there are no clinically approved radionuclide technologies for the management of HCC; however, several antibody-targeting agents are in preclinical development^24–27^.

Glypican-3 (GPC3) is a cell surface antigen highly expressed on HCC that has been used for a variety of targeted therapies including chimeric antigen receptor T-cell (CAR-T) therapy, vaccine and radionuclide therapy^28,29^. GPC3 is expressed on over 75% of HCC tumors and is not found on normal tissues^30,31^. GPC3 is variably expressed in HCC, and although highest cell surface expression is associated with de-differentiated tumors, it is also present on well-differentiated tumors and dysplastic nodules indicating it may be effective in identifying early neoplastic lesions^32^. It is therefore an attractive target for developing a theranostic platform which combines detection and treatment of both early and advanced disease.

Delivery of radioimmunoconjugates for a theranostic approach, including initial tumor identification and staging, subsequent targeted radioimmunotherapy, and assessment of treatment response has not been reported in HCC. To this end, our group developed GPC3-targeted radioimmunoconjugates using zirconium-89 (^89^Zr-αGPC3) for immuno-PET imaging and ytrrium-90 (^90^Y-αGPC3) for cytotoxic radioimmunotherapy to serve as functional agents for a theranostic approach^24,33^. In this proof-of-concept study, we test the utility of sequentially delivering radioimmunoconjugates to identify, treat and assess treatment response in a murine orthotopic xenograft model of HCC.

## Results

### Conjugated αGPC3 maintains binding affinity for GPC3

**Figure 1:**
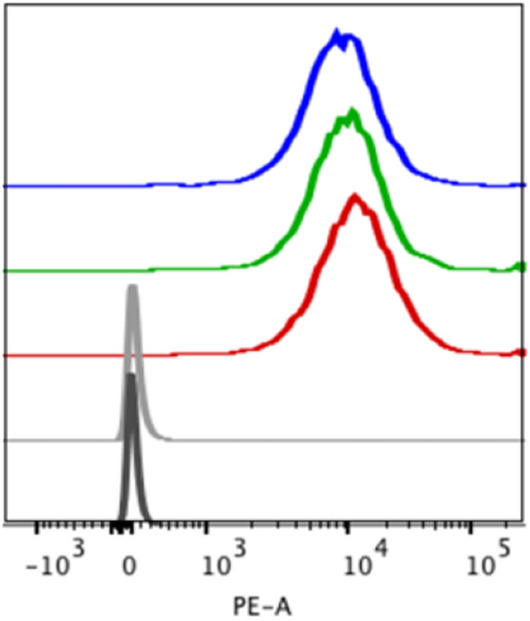
Conjugated αGPC3 maintains binding affinity for GPC3 *-*In vitro GPC3 binding assessed by flow cytometry on HepG2 cells with unconjugated (red), DOTA- (blue) and DFO- (green) conjugated αGPC3 compared to unstained (black) and secondary-only control (gray) antibodies.

Binding to cell surface GPC3 by unconjugated, DFO- and DOTA-conjugated αGPC3 was confirmed by flow cytometry (Fig. 1). Normalized geometric mean fluorescence for αGPC3-DFO and αGPC3-DOTA were significantly elevated compared to control. These validated conjugates were subsequently radiolabeled with ^89^Zr for immuno-PET studies and ^90^Y for RIT.

### ^89^Zr-αGPC3 immuno-PET reliably identifies small tumors and measures tumor volume

**Figure 2:**
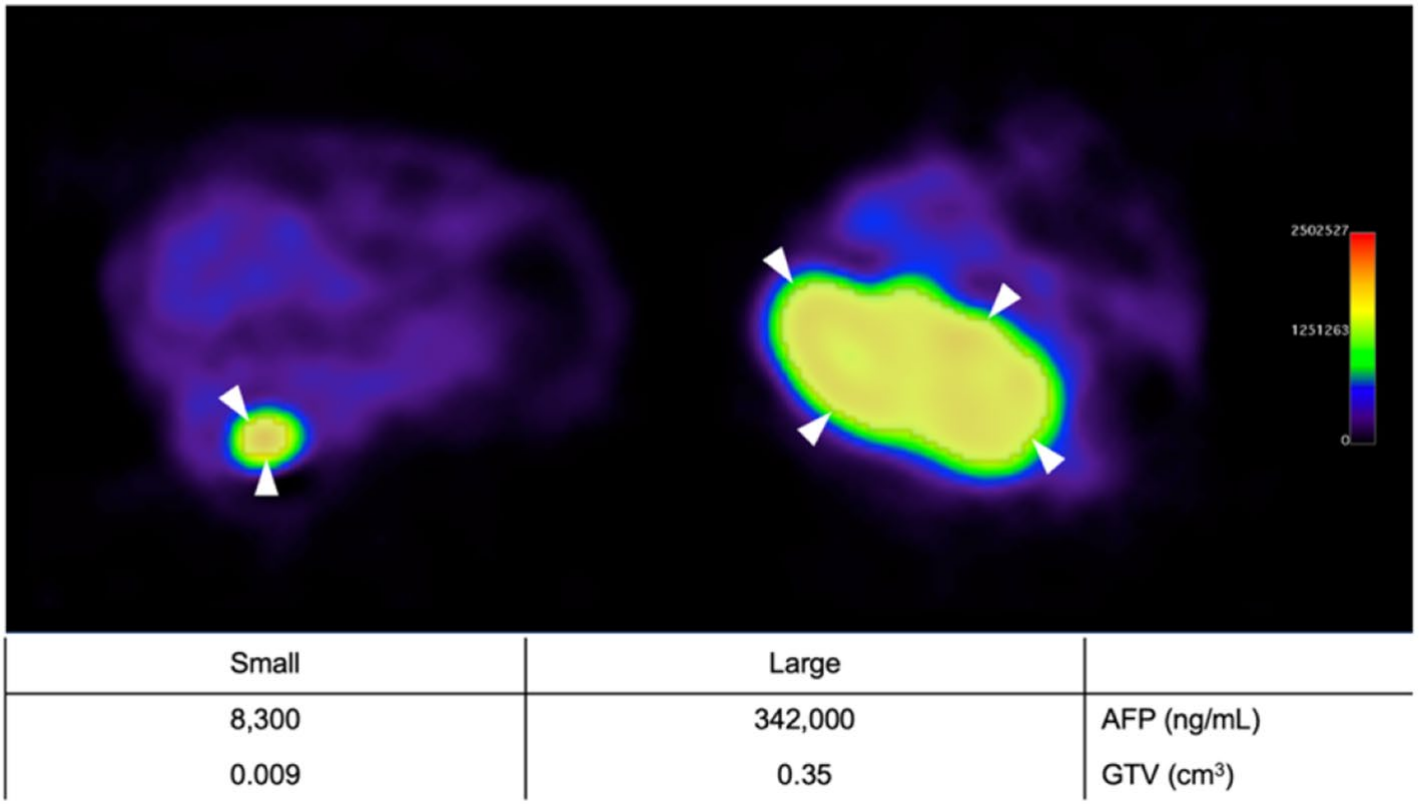
^89^Zr-DFO-αGPC3 immuno-PET identifies and measures volume of small and large tumors—Representative axial immuno-PET images of pre-treatment animals with small (left) and large (right) tumor with region of interest (shaded) contour set at 40% of maximum signal intensity (white arrowheads). Corresponding serum AFP and immuno-PET GTV listed in table below.

**Figure 3:**
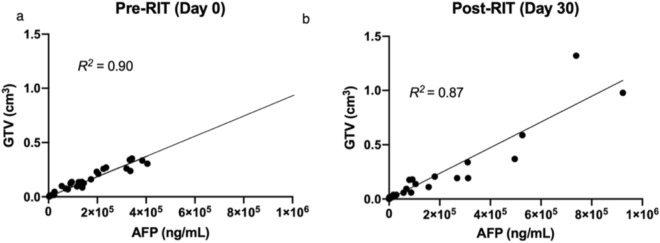
^89^Zr-αGPC3 immuno-PET measures tumor volume before and after αGPC3 exposure. (**a**) GTV measured by ^89^Zr-DFO-﻿αGPC3 immuno-PET correlates with tumor burden on day 0 (n = 29) and (**b**) 30 days after ^90^Y-DOTA-αGPC3 RIT (n = 27). Linear, least squares regression analysis without special handling of outliers or weighting was performed.

Orthotopic tumor establishment was confirmed with serum AFP measurement and IVIS bioluminescence imaging six weeks after subcapsular HepG2 cell injection. To stage tumors prior to RIT, 29 animals underwent ^89^Zr-DFO-αGPC3 immuno-PET imaging and GTV was measured along with serum AFP sampling. All animals demonstrated discrete localizations of increased PET intensity at the subcapsular injection site with minimal background signal (Fig. 2). Immuno-PET was sensitive in detecting both small and large tumors, with GTVs ranging from 0.009cm^3^ to 0.35cm^3^ . Individual animal immuno-PET GTV measurements strongly correlated with serum AFP concentrations (*R*^2^ of 0.90) (Fig. 3a), demonstrating its reliability in assessing tumor burden and measuring tumor volume. Maximum standardized uptake (SUV_max_) were calculated from PET images prior to RIT and ranged from 6 to 53.5 and did not correlate with tumor size (data not shown).

### ^90^Y-αGPC3 RIT decreases tumor burden

**Figure 4:**
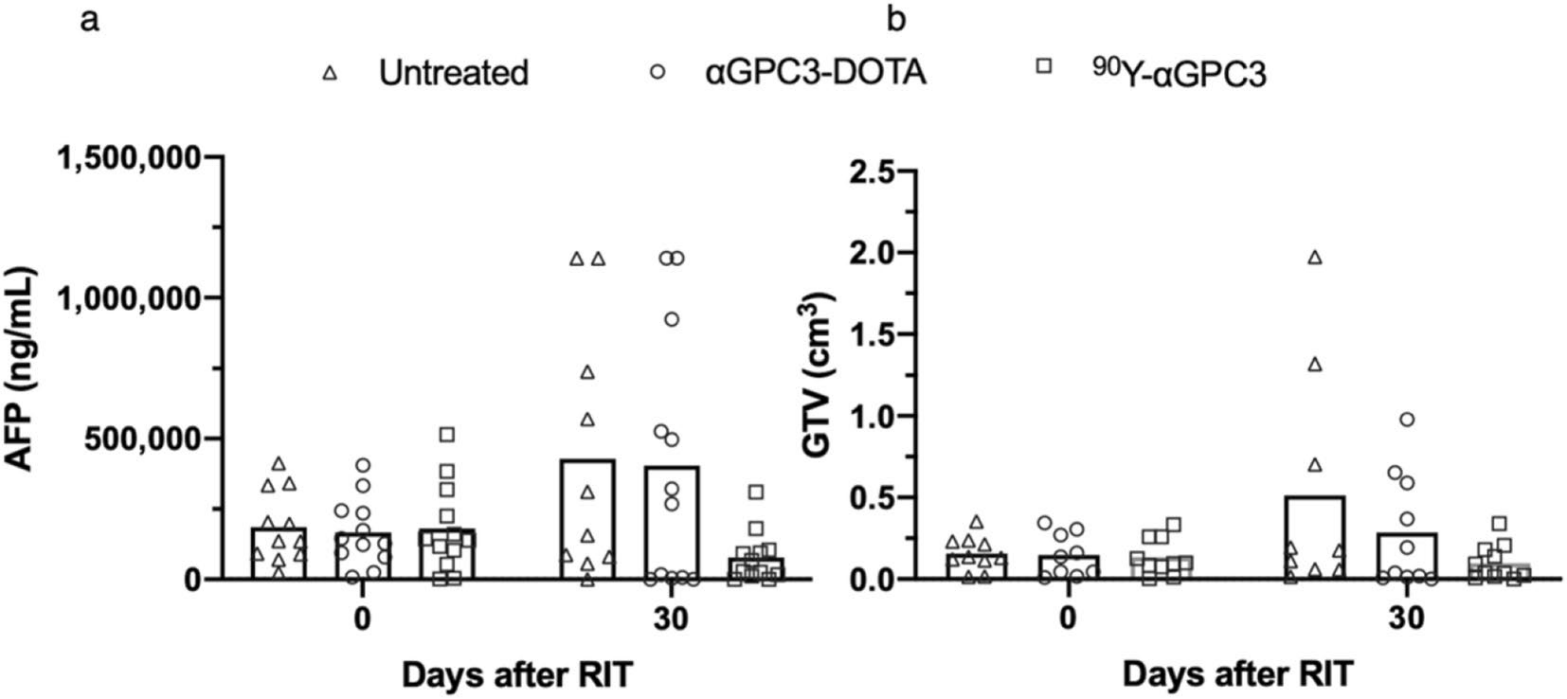
^90^Y-DOTA-αGPC3 RIT decreases tumor burden—(**a**) Serum AFP measurements in untreated (n = 11), non-radiolabeled αGPC3-DOTA treated (n = 12) and ^90^Y-DOTA-αGPC3 treated (n = 12) mice and (**b**) GTV measurements by ^89^Zr-DFO-αGPC3 immuno-PET in untreated (n = 10 pre-RIT, n = 9 post-RIT), non-radiolabeled αGPC3-DOTA treated (n = 9 pre-RIT, n = 10 post-RIT) and ^90^Y-DOTA-αGPC3 RIT treated (n = 10 pre-RIT, n = 11 post-RIT) mice before (day 0) and 30 days after therapy. Bar represents mean. Normality of data assessed via Shapiro Statistical analysis by One. Kruskal–Wallis with Dunn’s multiple comparison test was performed for pairwise comparisons. * denotes *p* < 0.05.

Two days after ^89^Zr-DFO-αGPC3 immuno-PET imaging, the animals received either 11.1 MBq ^90^Y-DOTA-αGPC3, αGPC3-DOTA alone by tail vein injection or no treatment. Tumor burden was not statistically different between groups prior to RIT, with mean serum AFP concentrations of 185,986 ± 127,154 ng/mL in the no treatment control group, 166,370 ± 119,541 ng/mL in the αGPC3-DOTA control group, and 180,613 ± 155,557 ng/mL in the ^90^Y-DOTA-αGPC3 group (p = 0.97). Pre-RIT GTV was 0.16 ± 0.10cm^3^ in the no treatment, 0.15 ± 0.13cm^3^ in the αGPC3-DOTA control groups, and 0.14 ± 0.12cm^3^ in the ^90^Y-DOTA-αGPC3 group (p = 0.96) (Fig. 4a,b).

**Figure 5:**
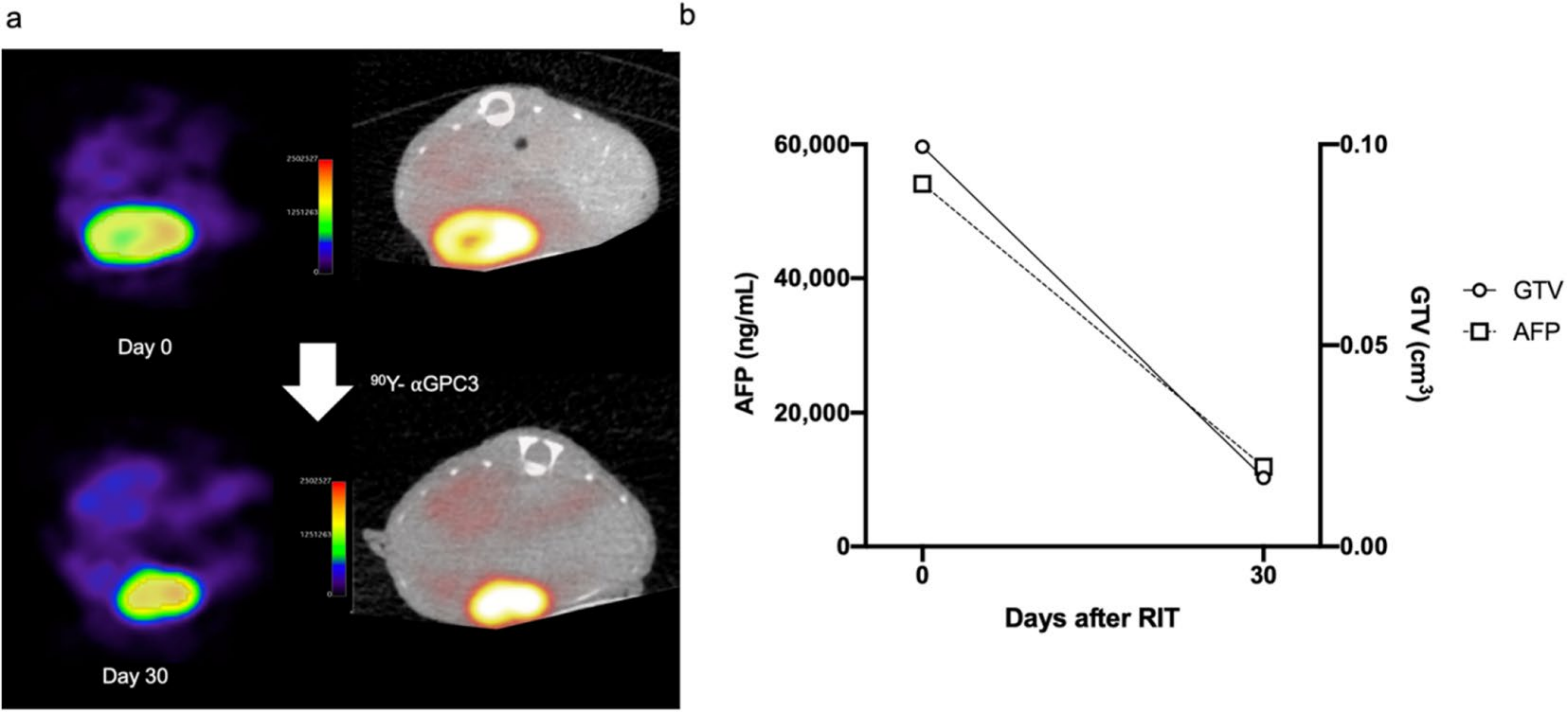
^89^Zr-DFO-αGPC3 immuno-PET evaluates treatment response after ^90^Y RIT. (**a**) Representative axial immuno-PET (left) and fused PET/CT (right) images from before (day 0) and 30 days after ^90^Y-DOTA-αGPC3 RIT. Shaded PET area represents isocontour at 40% maximum signal intensity. (**b**) Serum AFP and GTV of same animal before (day 0) and 30 days after ^90^Y-DOTA-αGPC3 RIT.

Thirty days after RIT injection, mean serum AFP was measured and animals underwent a second round of ^89^Zr-DFO-αGPC3 immuno-PET imaging. Mean serum AFP concentration was lower in the ^90^Y-DOTA-αGPC3 treatment group compared to both no treatment and αGPC3-DOTA control groups (Fig. 5a). Compared to pretreatment levels, mean serum AFP concentration decreased 57% to 77,834 ± 90,970 ng/mL, in the ^90^Y-DOTA-αGPC3 treatment group, and increased 150% to 428,221 ± 444,127 ng/mL, and 143% to 403,835 ± 446,544 ng/mL, in the no treatment and αGPC3-DOTA control groups, respectively. A significant reduction in serum AFP 30 days after ^90^Y-DOTA-αGPC3 RIT was observed in all but one animal. One untreated animal required euthanization prior to completion of the study due to large tumor size.

### ^89^Zr-αGPC3 immuno-PET evaluates treatment response after ^90^Y RIT

To examine the utility of ^89^Zr-DFO-αGPC3 immuno-PET in assessing treatment response, 29 animals imaged prior to RIT underwent a second round of immuno-PET imaging 30 days after RIT. Again, we observed discrete localizations of increased PET intensity in the left lobe of the liver in all imaged animals, indicating that serial exposure to our antibody does not limit binding of additional radiotracer doses, likely through replenishment of surface antigen (Fig. 5a). Similar to pre-treatment immuno-PET imaging, GTVs correlated with serum AFP concentration after therapy (*R*^2^ = 0.87, Figs. 3b, 5b), confirming its accuracy in measuring tumor volume after serial antibody exposure. No significant correlation between pre-treatment SUV_max_ and response to ^90^Y RIT was observed. Lower intensity PET signal was observed in the center of larger tumors, suggesting diminished conjugate delivery secondary to poor perfusion and/or tumor cell necrosis and loss of GPC3 surface expression.

Immuno-PET imaging with ^89^Zr-DFO-αGPC3 demonstrated an increase in GTV of 226% to 0.51 ± 0.70cm^3^, and 115% to 0.31 ± 0.35cm^3^, in untreated and non-radiolabeled αGPC-DOTA animals, respectively, and a decrease in GTV in ^90^Y treatment animals by 62% to 0.10 ± 0.10cm^3^, compared to pretreatment volumes, in a trend toward statistically significant differences between groups (*p* = 0.13) (Fig. 4b). Of note, due to limitations in imaging logistics during 2nd round, several randomly selected animals were not imaged by immuno-PET. Three animals in the no treatment control group were not imaged, two of them having large tumors as measured by serum AFP (570,000 and 1,114,000 ng/mL). Two animals in the non-radiolabeled αGPC-DOTA control group were not imaged, both of them found to have large tumors by serum AFP (322,000 and 1,114,000 ng/mL). One animal in the ^90^Y-DOTA-αGPC3 group was not imaged, with moderate sized tumor by serum AFP (95,000 ng/mL).

### ^90^Y-αGPC3 RIT induces tumor cell apoptosis

**Figure 6:**
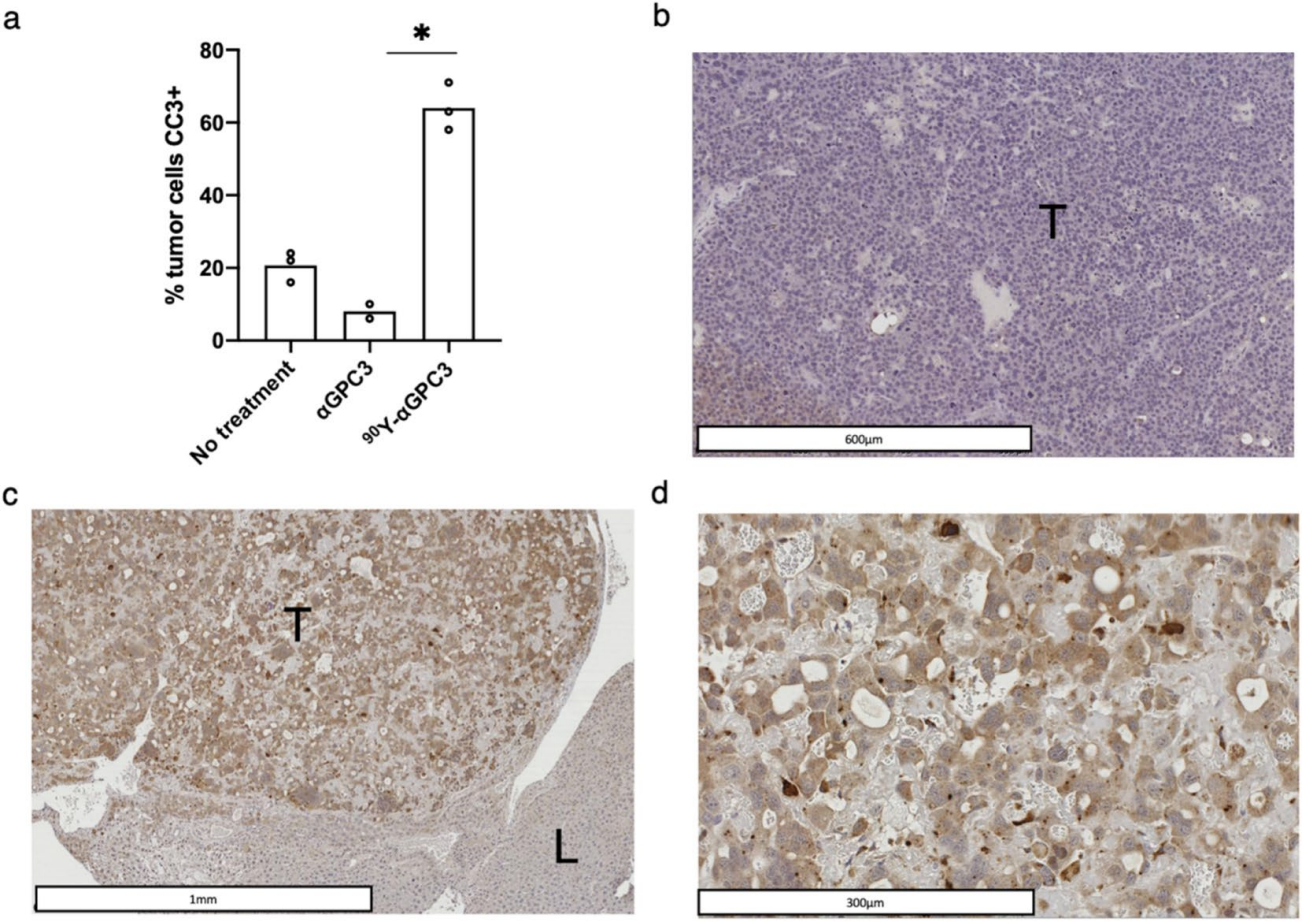
^90^Y-DOTA-αGPC3 RIT induces tumor cell apoptosis. (**a**) Quantification of intra-tumoral cleaved caspase-3 (CC3) expression in untreated, non-radiolabeled αGPC3-DOTA and ^90^Y-αGPC3 treated mice (*n* = 3, 2, 3). (**b**) Representative CC3 IHC of tumor (T) and normal liver (L) in untreated and (**c**, **d**) in ^90^Y-DOTA-αGPC3 treated mouse. Bar represents mean, symbol represents mean of each mouse evaluated. Statistical analysis by Kruskal–Wallis with Dunn’s multiple comparison test was performed for the depicted pairwise comparisons. *Denotes *p* < 0.05.

Select animals were euthanized after ^89^Zr-DFO-αGPC3 immuno-PET and livers were removed *en bloc* for histopathologic analysis. Immunohistochemical analysis of CC3, a marker of apoptosis, demonstrated significant increase in apoptosis in tumors after ^90^Y-DOTA-αGPC3 RIT, with 64% of cells expressing CC3, compared to 20% in untreated and 8% in αGPC3-DOTA control groups (Fig. 6a–d). CC3 expression was not observed in any surrounding normal liver parenchyma (Fig. 6b) confirming cytotoxic effect limited to tumors cells specifically targeted by ^90^Y-DOTA-αGPC3 RIT.

## Discussion

This preclinical study demonstrates the utility in combining ^89^Zr-DFO-αGPC3 and ^90^Y-DOTA-αGPC3 for detection and therapy in a specific theranostic approach to stage, treat, and assess response to treatment in an orthotopic xenograft model of HCC, highlighting the potential direct clinical relevance for patients with HCC. To test theranostic feasibility, we delivered our radiolabeled αGPC3 sequentially over time to assess efficacy in the setting of serial antibody exposure. Using ^89^Zr-DFO-αGPC3 immuno-PET, we reliably measured volume of sub-centimeter implanted tumors, which correlated well with markers of tumor burden indicating utility in initial clinical staging. ^90^Y-DOTA-αGPC3 RIT effectively reduced tumor burden with a decrease in serum AFP and ^89^Zr-DFO-αGPC3 immuno-PET tumor volume compared to control. Correlations between serum AFP and ^89^Zr-DFO-αGPC3 immuno-PET tumor volume remained strong after RIT suggesting ^89^Zr-DFO-αGPC3 immuno-PET may be useful in assessing response to therapy. These findings confirm the effectiveness of each radioimmunoconjugate in the presence of serial antibody exposure. If effective in patients with HCC, this target-specific diagnostic and therapeutic approach would represent a major advance over current technologies used in HCC that would specifically benefit patients with GPC3 expressing tumors.

This study validates the ^89^Zr-DFO-αGPC3 immuno-PET agent as a highly sensitive and specific molecular imaging agent for imaging GPC3-positive tumors. All animals with tumors identified by bioluminescent imaging and serum AFP measurement had detectable tumors by immuno-PET before and after RIT. ^89^Zr-DFO-αGPC3 immuno-PET detected sub-centimeter tumors, with tumor sizes ranging from 0.009 to 1.9cm^3^, and reproducibly measured volume using standardized segmentation technology^34,35^. Although there are several limitations associated with PET GTV quantification^36^, we were reassured to see a strong correlation between measured GTV and serum AFP before and after RIT. Further studies to validate the accuracy of this tumor volume quantification are currently ongoing.

We observed a reduction in tumor size after treatment with ^90^Y-DOTA-αGPC3. This effect was observed in animals exposed to antibody for PET imaging just a week prior, indicating that the antibody binding sites were not saturated or that bound sites were quickly recycled. The antitumor effect may be explained by antibody-dependent cellular cytotoxicity which has been observed with other αGPC3 antibodies targeting different epitopes, including GC33, which has been evaluated in clinical trials^37^. However, in our study, we did not observe a measurable antitumor effect in the non-radiolabeled antibody control group, indicating that this antitumor effect was due to the presence of targeted radiation. This targeted RIT is delivered systemically and may be an important new therapy for patients with advanced GPC3-expressing HCC.

This study is the first to report a combined PET/RIT approach to the treatment of HCC and represents an advance in the field towards a more personalized and targeted radionuclide approach. This contrasts with the current ^90^Y radionuclide therapy used in HCC which is delivered in microspheres via a transarterial approach. The antibody directed approach offers a systemically administered alternative and would benefit from GPC3 targeting. Although only applicable to GPC3^+^ tumors, these patients would be selected for therapy with the ^89^Zr-DFO-αGPC3 immuno-PET, increasing likelihood of treatment success. This theranostic platform for HCC complements the growing field of radionuclide theranostics for management of solid tumors. Since the 1980s, several theranostic agents were developed including those for colorectal cancer^38^, breast cancer^39^, renal cell carcinomas^40^, non-Hodgkin’s lymphoma^41^ and prostate cancer^42^. The most widely utilized agent is used in the management of neuroendocrine tumors with ^68^ Ga-DOTATATE PET for identification and ^177^Lu DOTATATE as PRRT^19,43^. These studies support the development of more theranostic platforms to personalize patient selection with targeted molecular imaging followed by targeted therapy.

There are several limitations to our preclinical study. Due to PET scanner availability, not all animals were imaged after RIT, including several control animals with high tumor burden. Although shown to be generally widely expressed on HCC, the extent of GPC3 expression varies and may limit the sensitivity of the tumor volume quantification and efficacy of RIT for all HCCs observed in clinical practice. We did not observe an association between SUV_max_ and response to radioimmunotherapy or tumor size in this study. In fact, a weak correlation was observed between higher SUV_max_ and smaller tumor size.This may be explained by reduced radiotracer uptake from insufficient perfusion or intratumoral necrosis in our model, which warrants investigation in future studies. Utilizing a cell line with high GPC3 expression in an immunocompromised xenograft does not adequately represent the complex human HCC tumor microenvironment and favors the function of our GPC3-targeting agents; however, this proof-of-concept study was important to demonstrate the potential of our platform. Additional experiments demonstrating the limit of GPC3 expression needed for detection and effective therapy with our theranostic platform are needed. Furthermore, although PET image segmentation is limited by PET technique and its partial volume effect, we still observed very strong correlations between tumor volume by PET and validated measures of tumor volume, particularly for smaller tumors. More investigations will be needed to assess the accuracy of PET outside this range.

## Conclusion

In this study, we report GPC3 targeted radionuclide delivery of ^89^Zr/^90^Y as a promising theranostic approach to detect, treat, and monitor treatment in an orthotopic xenograft model of HCC. By utilizing the ^89^Zr-DFO-αGPC3 immuno-PET imaging platform to assess the efficacy of ^90^Y-DOTA-αGPC3 RIT, we confirm the continued utility of our radioimmunoconjugates with serial antibody exposure, demonstrating feasibility of this theranostic platform as a future approach to improve diagnosis and treatment of patients with HCC.

## Materials and methods

### Cell lines and tissue culture

GPC3-positive HepG2-Red-FLuc HCC cells expressing *Luciferase* were purchased from PerkinElmer (RRID:CVCL5I98, Bioware, cat. no. BW134280) and were maintained as previously described^33^. Cells were cultured in a monolayer at 37 °C in Dulbecco’s modified Eagle Medium (DMEM; Gibco) supplemented with 10% fetal bovine serum (FBS; Gibco) in a humidified chamber with 5% CO_2_. Cells were grown until 70–80% confluent, detached with 0.25% trypsin and passaged into new cell culture flask per manufacturer’s instructions (PerkinElmer).

### Production of αGPC3 IgG1

As previously described, αGPC3 IgG1-producing hybridomas were generated through the Fred Hutchinson Cancer Research Center antibody core facility^44^.

### Conjugation of αGPC3 with deferoxamine (DFO) and 1,4,7,10-tetraazacyclododecane-1,4,7,10-tetraacetic acid (DOTA)

To demetallate the αGPC3 antibody for labeling with radiometals, it was concentrated to 6 mg/mL and then dialyzed against metal-free saline (150 mM NaCl, and 1 mM EDTA adjusted to pH 7 and passed over a Chelex 100 column) for 3 days at 4 °C with a minimum of three buffer changes a day. The day before the conjugation reaction, the antibody was dialyzed for an additional two buffer changes to replace the saline with demetallated HEPES buffer (50 mM HEPES (*N*-(2-hydroxyethyl)piperazine-*N*′-ethanesulfonic acid, 150 mM NaCl, and 1 mM EDTA adjusted to pH 8.5 and passed over a Chelex 100 column). In acid washed microcentrifuge tubes, the demetallated αGPC3 antibody was conjugated with 10 equivalents of either p-SCN-Bn-DFO or p-SCN-Bn-DOTA (both from Macrocyclics) as a solution at 10 mg/mL in DMSO. These reactions were allowed to run overnight at room temperature with gentle mixing. The reaction mixtures were then dialyzed against a metal-free citrate buffer (50 mM sodium citrate and 150 mM NaCl with pH 5.5) over 3 days at 4 °C followed by dialysis against 150 mM saline (pH 7.0) for another 3 days. Each buffer change contained Chelex resin to scavenge metals. The final conjugates were then stored in acid washed tubes at 4 °C.

### ^89^Zr-Labeling of αGPC3-DFO

Demetallated 2 M sodium carbonate was added to ^89^Zr to adjust the pH to 7.0–7.5. To this solution was added HEPES buffer at pH 7.0, followed by αGPC3-DFO, prepared as above. The mixture was then incubated for 2 h at room temperature. The labeled antibody was separated from unreacted ^89^Zr via a PD-10 column (GE Healthcare) eluted with PBS prior to analysis by HPLC) to verify radiochemical purity. Acid-washed vials and pipette tips were used for all steps. The radiochemical yield was 78% and the radiochemical purity was > 95%.

### ^90^Y-Labeling of αGPC3-DOTA

αGPC3-DOTA was radiolabeled with ^90^Y as previously described^33^. Acid-washed vials and pipette tips were used for all steps. The radiochemical yield was 90% and the radiochemical purity was > 98%.

### Flow cytometry

Flow cytometry was used to evaluate the in vitro binding of the αGPC3-DFO and αGPC3-DOTA conjugates. HepG2-Red-FLuc cells were resuspended in cold phosphate-buffered saline (PBS) at a concentration of 1 × 10^6^ cells/mL. One microgram of unconjugated αGPC3, αGPC3-DFO or αGPC3-DOTA was added to the cell suspension and incubated for 45 min at 4 °C. After incubation, samples were washed in cold PBS, and incubated with 1 μg of PE-labeled goat-α-mouse IgG1 secondary antibody (BD Biosciences, San Jose, CA) on ice for 30 min in the dark. Cells were then washed in cold PBS and were analyzed with a LSRII (BD Biosciences, San Jose, CA) using the FACS Diva software. A minimum of 10,000 cells were analyzed for each sample in triplicate. Data analysis was performed on the FlowJo software, version 8.8.6 (TreeStar, Ashland, OR).

### Orthotopic xenograft model

This study was performed in accordance with the University of Washington Office of Animal Welfare guidelines for the humane use of animals, and all procedures were reviewed and approved by the Institutional Animal Care and Use Committee (Protocol #4304-02) and was carried out in compliance with the ARRIVE guidelines^45^. The orthotopic xenograft model was generated as previously^33^. After a week of acclimatizing, approximately 2 × 10^6^ Luciferase-expressing GPC3-positive HepG2 cells in 25μL of Dulbecco’s modified Eagle medium containing 50% Matrigel (BD Biosciences) were injected into the subcapsular space of the left hepatic lobe. Six weeks after hepatic subcapsular cell injection, a 75 mg/kg intraperitoneal injection of VivoGlo luciferin (Promega) was administered and bioluminescent imaging was performed using an IVIS Lumina II system (PerkinElmer) to verify tumor establishment.

### Measurement of serum AFP

To monitor orthotopic tumor growth, whole blood was obtained from animals by submandibular bleed and collected in EDTA-coated Eppendorf tubes^33,46^. Serum was extracted from the fresh whole blood, then frozen and allowed to decay 10 half-lives (~ 27 days for ^90^Y, ~ 33 days for ^89^Zr) if found to be radioactive in accordance with the University of Washington Environmental Health and Safety policy. Frozen serum specimens were thawed on ice and the serum concentration of AFP was determined on the UniCel Dxl 800 Access Immunoassay System (Beckman Coulter) using an Access AFP Alpha-fetoprotein pack (Quest Diagnostics) and reported in nanograms/milliliter.

### In vivo ^90^Y radioimmunotherapy

HepG2 tumor bearing animals were assigned to one of three experimental groups based on serum AFP measurements to ensure comparable cohorts and were grouped in same cage (n = 12 in each group). Animals either received no injection, 70 μg αGPC3-DOTA without radioisotope or 70 μg DOTA-αGPC3 radiolabeled with 11.1 MBq (300uCi) of ^90^Y (Perkin Elmer), administered via tail vein injection. Thirty days after RIT injection, randomly selected animals were imaged by PET/CT and then all animals were euthanized for serum AFP collection to evaluate for early anti-tumor effect of RIT. Primary outcome measure was serum AFP and gross tumor volume assessed on immuno-PET. Randomly selected livers were harvested and placed in 10% (w/v) neutral-buffered formalin. Animals were euthanized if they did not meet standard body-conditioning scores set by our Office of Animal Welfare.

### Small-animal positron emission tomography

^89^Zr-DFO-αGPC3 imaging studies were performed using the Inveon PET/CT scanner (Siemens Medical Solutions USA, INC. Molecular Imaging, Knoxville, TN), which was calibrated for ^89^Zr. Whole-body PET and CT imaging was performed on animals anesthetized with 1–2% isoflurane anesthesia in 100% oxygen at 1L/min in a temperature-controlled bed with respiratory monitoring. Tumor-bearing animals were injected with 11.1 MBq (300uCi) of ^89^Zr-DFO-αGPC3 (~ 70 μg antibody) via the tail vein 1 week before (n = 29) and 4 weeks (n = 27) after RIT injection. Pre-RIT PET/CT imaging was performed on days 4 and 5 after ^89^Zr-DFO-αGPC3 injection and post-RIT imaging was performed on days 6 and 7 after injection due to equipment availability. Animals first had a 60-min PET scan followed by a CT scan, which enabled scatter and attenuation correction. Due to scanner malfunctions during post-RIT imaging, 10 animal scans were PET-only and therefore not corrected for scatter or attenuation. Due to limited imaging time on PET scanner, not all animals were imaged. Animals were randomly selected and were imaged before and after RIT.

### PET tumor volume and intensity measurements

PET images were reconstructed using ordered subset expectation maximization/shifted Poisson maximum a posteriori (OSEM3D/SP-MAP; 2 iterations 18 subsets) with a 256 × 256 matrix, target resolution of 1.5 mm, zoom factor 1.3, and corrections for scatter and attenuation. CT images were reconstructed using forward back projection with a Shepp-Logan filter, slight noise reduction, and appropriate beam hardening corrections. Maximum Standardized Uptake Values (SUV_max_) were determined using MIM software (MIM Software Inc., Cleveland, OH USA). Dose information for each mouse was entered as 8.51 MBq (0.230 mCi) ^89^Zr at 14:30 1/10/19, along with measured mouse weight and scan start time. Because two animals were present in each image, the process was repeated for the second mouse. Gross tumor volume (GTV) measurement and image analysis were performed with Horos software (Nimble Co., Annapolis, MD USA). Localizations of increased intensity in the midline upper abdomen as anticipated based on anatomy and surgical procedure were classified as tumors. GTV was measured using a combination of fixed thresholding and manual segmentation methods as previously described^35^. A fixed, relative threshold of 40% of the maximum signal intensity was set as the lower threshold for each growing region. For larger tumors with diffuse, heterogeneous uptake, manual visual segmentation was performed. The total and mean signal intensity of each volume of interest was obtained and was decay and dose-corrected. Since 10 animals did not have attenuation or scatter correction, GTV measurement was performed with images that were not attenuated or corrected for scatter to maintain consistency of measurements across cohort. All segmentation was performed by a user blinded to experimental conditions of each animal.

### Immunohistochemistry

Formalin-fixed, paraffin-embedded tissues were sectioned at 4 microns onto positively charged slides. Slides were de-paraffinized in xylene and rehydrated through graded ethanol. Heat mediated antigen retrieval was performed in 10 mM sodium citrate buffer (pH 6.0). Incubations were performed with cleaved caspase-3 (CC3) primary antibody (1:200) (#9661, Cell Signal Technologies) followed by host-matched secondary antibody (1:200), polymer reagent and color substrate. Secondary reagents were ImmPress Rabbit HRP (Vector Laboratories) and color development was performed using Quanto DAB brown kit (Fisher Scientific) followed by counterstaining with Harris hematoxylin (Sigma). High-resolution slide scans were obtained with Hamamatsu NanoZoomer Digital Pathology System up to 40× magnification. Positive cell detection of 20X high power fields (20–30 per tumor) was performed using QuPath (QuPath, RRID:SCR_018257) image analysis software for quantification of CC3 positive cells (24).

### Statistical analysis

GraphPad Prism (version 8.0.0, GraphPad Software, San Diego, California USA, RRID:SCR_002798) was used for statistical analysis. D’Agostino & Pearson normality test was performed to test for Gaussian distribution. Continuous variables were expressed as medians and means and compared by Student’s t-test or Mann–Whitney test depending on distribution. One way-ANOVA or Kruskal–Wallis with Dunn’s multiple comparison test was performed for multiple depending on distribution. Correlation was assessed via linear regression with goodness-of-fit determination (*R*^2^). In all cases, a *P* value of ≤ 0.05 was considered statistically significant.

### Ethics approval

This study was approved by the Institutional Review Board and the Institutional Animal Care and Use Committee at the University of Washington.
